# Hip fracture care and national systems in Israel and South Africa

**DOI:** 10.1097/OI9.0000000000000065

**Published:** 2020-03-23

**Authors:** Yoram A. Weil, Brian P. Bernstein, Sithombo Maqungo, Amal Khoury, Meir Liebergall, Maritz Laubscher

**Affiliations:** a Department of Orthopaedic Surgery, Hadassah Hebrew University Hospital, Jerusalem, Israel; b Orthopaedic Research Unit, Department of Orthopaedic Surgery, Groote Schuur Hospital, University of Cape Town, Cape Town, South Africa

**Keywords:** hip fractures, health systems, geriatrics

## Abstract

Despite the same latitude on earth, Israel and South Africa have a wide variety of healthcare systems and approaches. Israel is a developed country with life expectancy within the first decile of the modern world. South Africa is a developing country where available resources and health care varies greatly across the country. Israeli policy makers have realized in 1999 the importance of early surgery for hip fractures as the single most important factor contributing to decreased mortality. After an introduction of a newer reimbursement system in 2004, and public advertising of early hip fracture treatment as a quality tag for hospitals, in more than 85% of the cases patients are operated on early (within 8 hours) with a significant decrease in mortality. However, other issues such as patient preparation, rehabilitation, and prevention are still at their beginning. South Africa deals with significant challenges with high energy hip fractures in a younger population, although osteoporosis is on the rise in certain parts of the country. Due to limited resources and distances, time to surgery differs among hospital systems in the country. In public hospitals, a delay up to a week may be common, whereas in private hospitals most patients are operated early within 48 to 72 hours. Due to decreased life expectancy, arthroplasty is more aggressively used in displaced femoral neck fractures. Rehabilitation is mostly done within the families. Prevention and orthogeriatric teamwork are not being commonly practiced. Generally speaking, more attention to hip fractures is needed from healthcare funders.

## 1 Introduction

The Middle East and the African continent have a wide divergence of resources and health systems and treatment approaches. Israel and South Africa are good examples of this wide range of care and resources. Israel is a developed country with life expectancy within the first decile of the modern world, while South Africa is a country where available resources and health care vary greatly across the country. Hip fractures are one of the most common orthopaedic conditions throughout the continent, and hip fracture care similarly varies throughout Africa. This report seeks to describe hip fracture systems and care in Israel and South Africa.

## 2 Israel

### 2.1 The evolution of hip fracture care in the state of Israel

In 1999, a consensus meeting arranged by the Israeli Center for Technology Assessment in Health Care, the Ministry of Health (MOH), and the Medical Association took place In Israel.^[1]^ In this meeting, the data from 1998 was presented during which 5400 people sustained hip fractures. The first-year mortality rate among these patients was 14.1% if operated on early (within 48 hours) vs a 25.7% first year mortality rate in patients operated on after 48 hours. The conclusion and recommendation following the meeting that was all patients, other than those for whom there was a clear contraindication for surgical intervention, should be operated on as soon as possible after injury. However, this recommendation was not fully implemented until 2004.

In 2004, the Medical Governance of the Ministry of Health in Israel decided to use a diagnosis related coding payment system for hip fixation surgery.^[2]^ The payment system was unique in that it incorporated the time from admission to surgery into the formula determining hospital reimbursement. If surgery was performed after 48 hours, then this payment was reduced significantly. Besides the reimbursement paid by the service providers to the hospital, this compensation system allowed the hospital to pay the surgeons and operating room personnel for after-hours performance of hip fracture surgery. In a follow-up study done in 2011,^[3]^ there was an increase of early hip fracture surgery from 49.7% to 67% within 2 years, as well as a reduction in total length of stay. Furthermore, there was a 35% reduction in mortality in patients undergoing fixation and a 30% mortality reduction with patients undergoing arthroplasty. A study performed in a level I trauma center demonstrated a mortality reduction that was sustained even for patients who were significantly older and with more comorbidities^[4]^ following the implementation of the reimbursement system. However, despite this new reimbursement system, hip fracture care did not fully evolve. In 2011, a survey conducted by the Israeli MOH demonstrated that the rise in early surgery was not uniform, with some centers approaching an early surgery rate of only 45%.^[5]^

In 2013, as a part of a new tactic the Israel MOH designated the proportion of patients operated within 48 hours as a part of a new “quality indicator.”^[6]^ Since 2016, the proportion of early surgery at each hospital has been published online^[7]^ and in the public newspapers annually. Consequently, since 2017, the goal of 85% timely hip fracture surgery has been achieved nationwide.

## 3 Current practice in Israeli hospitals, regarding key-points in hip fracture care

Israel is a small country with a population of 9,000,000, but carries significant differences among centers countrywide. To better understand practice patterns in Israel, leading trauma and orthopaedic surgeons from 6 level I centers and 4 regional trauma centers across the country were interviewed. The interviews dealt with the following topics: descriptions of dedicated clinical pathways for preparing hip fracture patients;^[8,9]^ management of pre-existing anticoagulant therapy; and strategies for treating trochanteric and femoral neck fractures. Below are the description of the interviews.

### 3.1 Clinical pathway and preparation for surgery

Despite the accumulating evidence regarding the benefit of a dedicated ortho-geriatric team and “fast-track” preparation for surgery,^[10,11]^ there are no well-adopted protocols, with the exception of 2 level—I trauma centers in Israel. The orthopaedic residents in most cases prepare the patients and consult internists if needed.

### 3.2 Policies for patients on anticoagulants

One of the major causes for delays worldwide for early hip fracture surgery is the prescription of anticoagulant therapy. Emerging evidence, though limited, demonstrates no adverse effects in operating on these patients early.^[12,13]^ In 3 level I trauma centers and 1 level II trauma center—patients’ anticoagulant therapies did not commonly cause delays to surgery. In the rest of the centers, a delay of 24 to 36 hours for surgery was common.

### 3.3 Choice of implants in intertrochanteric fractures

The trend for transition to intramedullary nailing for intertrochanteric fractures continues in Israel. Only 2 level I centers have strict guidelines for the use of a dynamic hip screw in stable (AO/OTA 31A1) fractures. The rest of the centers utilize intramedullary nails in 80% to 100% of patients with intertrochanteric fractures. Only in 3 level I trauma centers are intertrochanteric fixation surgeries supervised by attending surgeons.

### 3.4 Policy regarding arthroplasty for displaced femoral neck fractures

The most conservative centers (3 trauma centers and 1 regional trauma center) still use bipolar hemiarthroplasty for elderly patients and total hip arthroplasty for physiologically young (60–70) and active patients. However, increasing percentages of total hip arthroplasty are seen among centers in Israel with a varying degree of indications in patients—ranging from either independent outdoor ambulator, to any outdoor ambulator, to all the patients. There is a trend in hospitals to use cementless arthroplasties in most cases.

## 4 South Africa

### 4.1 Funding and access to care

South Africa has a 2-tiered medical system. An extensive network of private hospitals provides all aspect of medical care, including emergency medical and surgical care, to those with private medical insurance. Only 16% of the population is privately insured.^[14]^ The majority of South African patients are therefore reliant on public health care which is grossly under resourced. Funding in the public sector is not event-based and the national health budget is distributed via provinces to individual hospitals.

The access to care greatly varies between regions within the country. Overall, South Africa has 1.63 orthopaedic surgeons per 100,000 population, which is well below the World Health Organization target of more than 5. This is further aggravated by the fact that 95% of orthopaedic surgeons work in private hospitals and are concentrated in major cities. Therefore the density per uninsured population is even lower (0.36 per 100,000) and in some rural provinces there are no qualified orthopaedic surgeons in the public sector.^[14]^ The majority of orthopaedic care in rural areas is provided by general practitioners.

### 4.2 National guidelines and targets

Injury is a leading cause of death and morbidity in South Africa, representing 8% of all-cause mortality. There are no national guidelines or targets for the management of injuries. There is a national focus on the prevention and treatment of communicable diseases like HIV, tuberculosis, and malaria.

### 4.3 Incidence

A significant portion of hip fractures in South Africa are due to high-energy mechanisms. Gunshot-induced fractures also contribute with some major trauma centers treating more than 20 gunshot hip fractures per year.^[15,16]^

Fragility fractures occur at younger ages, with more younger people (under 60 years old) affected compared with other regions. Osteoporotic hip fractures in black South Africans are associated with a high HIV prevalence and antiretroviral therapy. Both the HIV virus and antiretroviral treatment affect bone metabolism and are thought to increase the risk of secondary osteoporosis and therefore fragility fractures.^[17]^

Historically, it was believed that the incidence of osteoporosis and hip fractures is lower in black Africans compared with Europeans. More recent studies suggest that the incidence of osteoporosis in this group is increasing^[18]^ and fragility fractures may occur more commonly than previously thought.^[19]^ The incidence of osteoporotic hip fractures in Southern Africa, however, remains lower than global averages.^[18]^

The average life expectancy in South Africa varies from 61.1 years for males to 67.3 years for females.^[20]^ However, in sub-Saharan Africa at age 60 years, life expectancy is more than 14 years. This suggests that individuals who survive early adversities face a long period of old age.^[17]^ Sub-Saharan Africa now has twice the number of older adults (age over 60 years) than northern Europe.^[17]^ With osteoporosis on the rise, there is a large predicted increase in fragility fractures.

### 4.4 Preoperative and perioperative care

There are guidelines in place for the work-up for osteoporosis in patients with fragility hip fractures in South Africa.^[21]^ However, very few public hospitals have access to a geriatric medicine service, and the majority of hip fracture patients are managed solely by the orthopaedic team. A recent publication at a major public trauma hospital in South Africa found that no patients with a fragility fracture of the hip were comanaged by a geriatrician, and only 2% had an adequate work-up and initiation of treatment for osteoporosis, despite the fact that more than 15% of the patients in the series had a second fragility fracture. This led to the establishment of an orthogeriatric fracture liaison service at the unit.^[22]^

### 4.5 Time to surgery

There are no national guidelines for the time to surgery. At private hospitals, international targets of surgery within 48 to 72 hours of injury are mostly achieved.^[23]^ Select public hospitals meet these targets with a mean time to surgery of 49 hours.^[22]^ This, however, is not the case in the majority of public hospitals where major delays to surgery of more than 7 days are common.

### 4.6 Surgical strategies

Due to differences in life expectancies, local pathologies, and injury mechanisms, it is not feasible to utilize guidelines from more developed countries. With selected displaced intracapsular neck of femur fractures, South African surgeons are often more aggressive with arthroplasty in younger patients. This is due to the decreased life expectancy of patients, as well as the high failure rates with open reduction and internal fixation and the poorer outcomes with failed osteosynthesis.

### 4.7 Protocols for mobilization and rehabilitation

In South Africa, national health and social systems that facilitate long-term care do not exist. Family members take responsibility of the care of older relatives.

### 4.8 Protocols for falls prevention and bone health screening

There are no national programmes for the prevention of falls and bone health screening. Osteoporosis treatments, like bisphosphonates, are also not generally available in public health care.

## 5 Conclusion

The evolution of hip fracture treatment in Israel follows the rest of the industrialized countries and, in many respects, was ahead of its time; in 1999 and 2004, the recognition of the impact of early surgery and mortality were internalized by policy makers, and by 2019 more than 85% of hip fracture patients received early surgery, with a national 1-year mortality of less than 19%. Despite that, the country's health system has to improve in preoperative management and preparation, as well as secondary prevention. A different picture is seen in South Africa, where high-energy hip fractures are more commonly seen. Injury prevention as well as treatment of osteoporosis is required. Fragility fractures of the hip are on the increase in sub-Saharan Africa and should receive more attention from healthcare funders.
